# The Role of Gut Microbiota and Mucin Barrier in the Pathogenesis of Colorectal Cancer

**DOI:** 10.3390/cimb48010016

**Published:** 2025-12-23

**Authors:** Yifu Chen, Yunhua Xu, Xiong Li, Siming Wu, Hong Long, Guang Fu, Shuai Xiao

**Affiliations:** 1 Institute of Clinical Medicine, The First Affiliated Hospital, Hengyang Medical School, University of South China, Hengyang 421001, China; 20175761218@stu.usc.edu.cn; 2 Department of Gastrointestinal Surgery, The First Affiliated Hospital, Hengyang Medical School, University of South China, Hengyang 421001, China; 17769389680@163.com (X.L.); wsiming2025@163.com (S.W.); longhong@usc.edu.cn (H.L.); 2021010066@usc.edu.cn (G.F.); 3 Department of Pharmacology, School of Pharmacy, Tongji Medical College, Huazhong University of Science and Technology, Hang Kong Road 13, Wuhan 430000, China; xyh940707@163.com

**Keywords:** CRC, microbiota, mucus barrier, mucin, dysbiosis

## Abstract

Colorectal cancer (CRC) is one of the most common and fatal malignant tumors globally, and its development is increasingly related to the gut microbiota. Despite its effect on CRC having been extensively researched, the intestinal mucus barrier, which forms a fundamental link between the host tissues and gut microbes, is seldom discussed. A double-layered barrier, mainly formed by MUC2 mucin, isolates the outside world from epithelial cells to maintain intestinal homeostasis. Furthermore, it is subjected to a dynamic impact of microbial activity. Now, increasing evidence shows that mucus barrier disruption driven by certain gut microbes is an early event in the development of CRC. This review first introduces the structure and function of the colonic mucus barrier and then discusses how gut microbiota in different areas promote the development of CRC by disrupting the mucus layer. Finally, we examine translational opportunities for exploiting microbiota–mucus barrier interactions in CRC therapy.

## 1. Introduction

CRC is one of the top three most common and fatal cancers worldwide. The development of CRC is a complex process influenced by genetic, environmental, and lifestyle factors [1,2]. The gut microbiota is also called a huge “microbial organ” within our body, and it has been increasingly acknowledged among many other elements leading to illnesses. High-throughput omics technologies confirmed that there is considerable dysbiosis in CRC patients, which is commonly accompanied by abnormal proliferation of pro-inflammatory and genotoxic bacteria [3].

It is crucial to discuss the role of intestinal mucus as a barrier for gut bacteria when examining how germs cause illness. In normal circumstances, colorectal epithelial goblet cells are constantly secreting mucins to form a thick inner layer and a thin outer layer, which covers the intestine. The mucus barrier also makes it impossible for most bacteria and their harmful components that are present in the intestinal lumen to invade the epithelial cells directly, and at the same time, it is also a communication platform to communicate with the intestinal immune system and maintain intestinal homeostasis [4,5]. Disruption of this barrier has been widely shown to be a crucial step in the occurrence and progression of many forms of intestinal inflammatory disease and CRC [6].

Noteworthy is the intertwining and interdependence of the gut microbiota’s functions and those of the mucus barrier. The human gastrointestinal tract is home to a very dense microorganism population, and their collective genomes are acclaimed to be the human’s “second genome” [7,8]. Specific pathogenic bacteria, such as *Fusobacterium nucleatum* (*F. nucleatum*), *Escherichia coli* (*E. coli*), and *Bacteroides fragilis* (*B. fragilis*), exhibit aberrant colonization, which is linked to the progression of CRC [9,10,11]. The mucus layer represents the interface between the host and microorganisms. It serves not only as a habitat for commensal bacteria, which are nourished by the host, but also as a crucial barrier that protects against pathogenic invaders [12].

Therefore, we propose that the “Gut microbiota-mucus barrier functional unit” is the inevitable course of CRC development and progression and a promising candidate for new CRC prevention and treatment strategies. But existing studies mostly handled gut microbiota and mucus as somewhat independent factors; so they did not totally show how these two things work together and change over time when CRC is happening. Therapies modulating gut microbiota hold promise, but due to the lack of a systematic understanding of the functional axis, more accurate diagnostic and therapeutic strategies are hindered [13,14,15]. This review aims to clarify the “gut microbiota-mucus barrier” as a functional unit in CRC development and therapy. We will explore these three aspects in detail: gut microbiota and mucus composition and functions, gut microbiota and mucus dynamic equilibrium under normal conditions, how dysregulated gut microbiota and mucus lead to CRC proximally and distally via barrier breach and genotoxicity, new diagnostics and therapies for CRC focusing on gut microbiota and mucus, and the translation of those ideas. A systematic examination of such a key interactive interface has considerable theoretical significance and wide-ranging clinical applications.

## 2. Host–Microbe Interface: Colonic Mucus Barrier and Microbiota Ecosystem

### 2.1. The Mucus Barrier: A Dynamic, Layered Defense System

The colonic mucus barrier is primarily the interface between the abundant luminal microbes and the epithelial surface. This vital defense is mainly made and sent out by goblet cells, which form a double-layered structure with special job duties [16]. The innermost layer is thick and sticks to our skin; so it acts like a shield from the outside world, keeping the insides relatively germ-free all the time, even when healthy—nothing from outside can go directly on your skin or your eye [5]. Conversely, the outside is loose and provides a niche for the complex gut microbiota, acting as a repository for biochemical mediators, which are abundant in carbohydrates, antimicrobial peptides, and other host-derived factors [17]. The ability of a mucus layer to function as an effective barrier at a molecular level is contingent on two main groups of proteins—secreted proteins and transmembrane proteins.

Secreted proteins such as MUC2 and MUC5B are mainly produced by goblet cells via the classical secretory pathway (endoplasmic reticulum, Golgi apparatus, secretory vesicles) and secreted to the intestinal lumen, making up the bulk of mucus. Also, they are the main functional components of mucus that mainly contribute to the viscoelasticity of the mucus layer [18,19,20]. MUC2 is the most abundant secreted mucin found in colonic mucus, and its polypeptide backbone consists of characteristic tandem repeat sequences, the PTS domain, which is rich in proline, threonine, and serine. This is the main site of O-glycosylation [18,21]. Between and at the termini of the PTS domains are cysteine-rich segments that crosslink via disulfide bonds to form massive polymeric networks that MUC2 forms to constitute the mucus layer scaffold [22,23,24,25]. Mucins’ function greatly depends on their post-translational modifications, especially N-glycosylation and O-glycosylation. Among them, N-N-glycosylation plays a vital role in the early processing of mucin peptides to allow proper folding and dimerization of MUC2 [26]. In contrast, O-glycosylation happens mostly inside the PTS domain, and it forms and keeps the three-dimensional gel form of mucins, which is important for its viscoelasticity and protection from proteolysis [25,27,28]. In addition to secreted mucins, antimicrobial peptides (including alpha-defensins and lysozyme) secreted by Paneth cells also exist in the mucus layer. This provides a chemical defense to the barrier, directly killing or inhibiting bacteria, along with secreted proteins as a physical and chemical barrier to pathogens [29].

Transmembrane proteins are embedded in the apical membrane of intestinal epithelial cells, contributing to the formation of the glycocalyx on the surface of intestinal epithelial cells, located between the mucus and the cell membrane [30]. This group of proteins includes transmembrane mucins (MUC1, MUC3, MUC4, MUC12, MUC13, MUC17), which, together with tight junction proteins (claudins, occludin, zonula occludens proteins, and junctional adhesion molecules (JAM)), control intestinal permeability [31,32]. The extracellular domains of transmembrane mucins are also modified with O-glycans that act as sensors for changes in the external environment, such as bacteria, and trigger intracellular signaling to alter immune responses and cellular behavior [33].

In a short period of time, such as a few seconds or minutes, this dual barrier is not static, and it is in a state of dynamic equilibrium. The outer layer derives from the inner one by way of proteolysis; this procedure allows MUC2 polymers to stretch, though how it is done precisely has not been figured out yet [5,34,35]. This process is important for maintaining a physical barrier by intestinal epithelial cells and preventing pathogens from colonizing [5,18,36].

### 2.2. Gut Microbiota: Stable Ecological Network

The colonic environment is a low-oxygen environment with slow transit time, which provides a good habitat for anaerobic bacteria, and the bacterial community of the human body is the most concentrated and complex in the colonic environment [37]. This community is mainly composed of firmicutes and bacteroidetes, and the main representative genera include *Prevotella*, *Eubacterium*, and *Ruminococcus* [37]. This ecosystem performs functions that are very important for the health of the host, one of which is the fermentation of dietary fibers that result in the production of short-chain fatty acids (SCFAs), such as butyrate, acetate, and propionate. Butyrate, especially, is the primary energy supply for colonocytes and is crucial for improving the intestinal barrier, regulating immune responses, and maintaining intestinal homeostasis [11,38,39]. In addition, a stable and varied microbiota sets up a strong ecological network that stops pathogen colonization by competing and helps to restore balance after inflammation [40].

In this community, certain bacteria have developed specific adaptations to live at the mucosal interface. Take some bacteroides species and *Akkermansia muciniphila* (*A. muciniphila*), for example; they all have a huge group of glycoside hydrolases that allow them to eat the mucus glycans for nutrition and break down mucus [41]. Other commensals, including many firmicutes, are good butyrate producers that can help with barrier integrity directly and indirectly [42].

## 3. Mucin Dysregulation and Colorectal Carcinogenesis

During colorectal carcinogenesis, the expression and function of certain mucins undergo major modifications that promote tumor initiation and progression. Therefore, we focused on MUC5, MUC6, MUC16, and MUC20, which were typically expressed in CRC, and compared them with MUC1, MUC2, MUC4, MUC5AC, and MUC6, which are considered to be related to CRC by the mainstream [43].

### 3.1. MUC1

MUC1 is a highly glycosylated transmembrane mucin that is poorly expressed in the normal colon, but it is still involved in the mucus barrier, and this transmembrane mucin is highly overexpressed in CRC tissue [44]. MUC1 deficiency decreases tumor development by inhibiting inflammation, and increased expression of MUC1 lowers the abundance of CD8^+^ T lymphocytes, drives colonic tumor macrophage IL-6 release, and activates the transcription factor 3, causing CRC [45]. Moreover, NG-MUC1 is a mucin complex, and its structural domains may form a hydrophilic barrier to resist drug penetration and cause drug resistance [46].

### 3.2. MUC2

MUC2 is the main product of goblet cells, and it makes up the two-layered mucus of the colon, sterile on the inside and a microbial home on the outside, the main part of the mucus barrier [47]. It is often markedly down-regulated or completely absent in non-mucinous CRC, and the down-regulation of this may be related to the reduction in goblet cells, causing barrier damage, bacterial invasion, and exacerbated inflammation. In 20 normal colonic mucosa and 139 advanced carcinomas, Wang et al.’s study showed that low MUC2 expression may also be significantly associated with lymph node metastasis, poor cell differentiation, and advanced CRC stage [48]. But in the mucinous colorectal cancer (MAC) subtype, MUC2 expression is very high, which means a lot of mucin is made outside the cells. Current research has indicated that MAC is strongly linked to chemoresistance [49].

### 3.3. MUC4

MUC4 is generally expressed on goblet cells and epithelial cells of the colon and rectum. It has a big tandem repeat core, a nidogen region, an adhesion-linked region, a von Willebrand factor region, and three extra EGF regions [50,51]. Research has shown that MUC4 is linked to the MAPK pathway, PI3K-AKT pathway, JAK-STAT pathway, cell cycle, WNT pathway, and mTOR pathway, and it greatly impacts the prognosis of CRC [52].

### 3.4. MUC5

MUC5 contains MUC5AC and MUC5B; the former is secreted by gastric goblet cells, and the latter is mainly produced by respiratory epithelial cells. Both are minimally expressed or not expressed at all in normal colorectal cells. However, CRC cell lines show that MUC5AC is highly expressed in SW620 cells and MUC5B is highly expressed in LS174T cells [53,54]. The research found that MUC2 and MUC5 have the same chromosomal location (11p15.5), and the poor prognosis mechanism is also related to MUC2 [55]. Notably, abnormal MUC5AC expression is related to MSI and is undifferentiated [56].

### 3.5. MUC6

MUC6 is also secreted by gastric epithelial cells, and it is the main component of gastric mucus. An extensively glycosylated protein backbone, it is scarcely expressed in CRC [57]. Similarly, it is highly expressed in the LS174T CRC cell line [58]. However, it has been shown that the current data suggest that MUC6 is associated with a favorable prognosis in stages II and III of CRC [59].

### 3.6. MUC16

Like MUC1, MUC16 is a membrane-associated mucin with a very glycosylated extracellular domain [60]. It was first recognized as a biomarker of ovarian cancer, CA125, and it is currently the largest known mucin [61]. In both in vivo and in vitro studies, MUC16 overexpression has been consistently correlated to a poor prognosis in patients. MUC16 activates the JAK2/STAT3 signaling pathway mechanistically via direct binding to JAK2 [62].

### 3.7. MUC20

MUC20 is a transmembrane protein that is commonly seen in endometrial carcinoma and kidney-related disease [63,64,65]. According to the existing literature reports, Xiao et al. constructed tissue microarrays (TMAs) from 150 paraffin-embedded primary CRC tumor samples and adjacent normal tissue samples (ANCT) [66]. They found that MUC20 was expressed much more in CRC tissues than in the adjacent normal tissues, and its expression was related to recurrence and a poor prognosis. MUC2 can also enhance the migration and invasion ability of CRC cells. Currently, it is believed that the mechanism by which MUC20 overexpression plays a role in CRC may be related to shRNA, MMP-2, MMP-3, and E-cadherin [66].

## 4. Microbial Disruption of Mucosal Homeostasis and Carcinogenic Mechanisms in CRC

**Figure 1:**
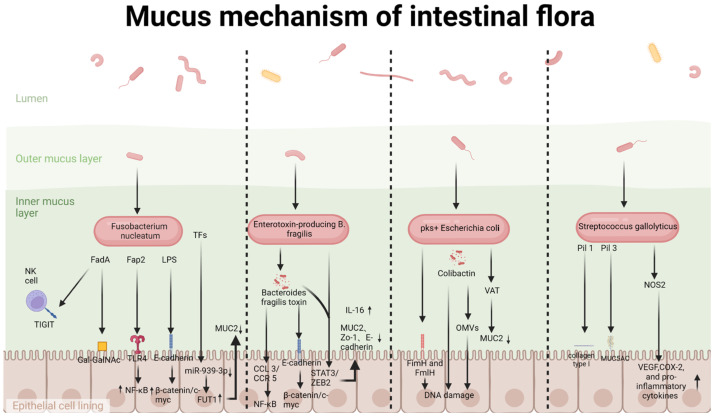
Mucus mechanism of intestinal flora. This schematic illustration summarizes the mucin-related oncogenic mechanisms of four key gut microbiota species implicated in colorectal carcinogenesis.

The gut microbiota displays notable spatial heterogeneity throughout the colonic axis, and distinct microbial communities can be observed in proximal and distal CRC (Table 1). While many commensal bacteria have a basal ability to degrade mucin for nutrient acquisition, in CRC this is dysregulated. More critically, some pathogens have evolved to actively manipulate the host’s mucin expression profile beyond simply consuming mucins, creating a tumor-permissive environment. We first outlined how the gut microbiota disrupts the mucus barrier. Subsequent analysis of differences in microbes between proximal and distal CRC tissues identified four bacterial species that are significantly associated with specific genetic changes in mucin genes (*F. nucleatum*, *Enterotoxigenic Bacteroides fragilis (ETBF)*, *Colibactin toxin-producing Escherichia coli* (*pks^+^ E. coli*), and *Streptococcus gallolyticus* (*S. gallolyticus*)) at a genetic level (Figure 1). These pathogens disturb the mucosal homeostasis in both direct and indirect ways.

### 4.1. Key Mechanisms of Mucus Barrier Disruption

#### 4.1.1. Structural Compromise of the Physical Barrier

The classic mucin-eating bacteria *A. muciniphila* feeds on mucin, serving to maintain gut homeostasis. Degradation, chiefly due to its secretion of glycoside hydrolases (GHs), such as GH33 (sialidase), GH16 (endo-β-galactosidase), and GH89 (a sialidase-like protein) [76]. The ability to produce these mucolytic glycoside hydrolases is not specific to *A. muciniphila*, as the recently described mucin-degrading genus *Alistipes* also harbors GHs that enable the degradation of the mucus layer [77].

In addition to these commensal degraders, certain pathogenic bacteria actively attack and compromise the mucus barrier, secreting virulence factors that target and degrade the main structural components of the mucus barrier. An enzymatic assault results in a thinner and more porous mucus layer, leading to a loss of its barrier function and allowing luminal microorganisms that are normally restricted to the outer layer to come into direct contact with the epithelium. *A. muciniphila* itself produces various proteins capable of degrading mucins. And one such example is the Amuc_1434 enzyme, which belongs to the aspartic protease family; it was found to enhance the adhesion to MUC2 high colon cancer cell line LS174T and also degrade MUC2 within the mouse colon [78]. A specific pathogen is the VAT protease produced by *pks+ E. coli*. It degrades the MUC2 mucin to allow bacteria to penetrate the mucus and reach epithelial cells. This activity strengthens the ability of *pks+ E. coli* to colonize the intestinal mucosa and thus increases its pro-carcinogenic activity [79].

#### 4.1.2. Alterations in Mucous Chemical Composition

Bacterial infection can interfere with normal host cell processes, causing abnormal patterns of mucin glycosylation. Carcinogenic bacteria mainly cause these changes by an increase in the branching of complex and hybrid N-glycans; an increase in the expression of sialyl Lewis antigens; a shortening of O-glycan chain lengths; and an increase in core fucosylation [80]. Abnormal glycosylation patterns don’t only have sway over rheological traits of mucus; they also expose hitherto hidden new bacterial binding epitopes that were covered up by sugars, thereby making more anchor points for pathogens to bind to and settle in. For example, *Porphyromonas*-derived SCAF can lead to expression of specific mucin O-glycans that are associated with epithelial differentiation in Caco-2 colon carcinoma cells [81]. This might be very important for making mucus.

#### 4.1.3. Reprogramming of Mucin Gene Expression

Carcinogenic microbiota can make a complete alteration in the expression situation of mucin genes for the host, and it can also activate some signaling pathways. A common tactic used by these microbes is to suppress the protective gel-forming mucins like MUC2. This transcriptional reprogramming is often brought about by bacterial metabolites such as SCFAs, BAs, and Trp metabolites. Evidence points to SCFAs, especially butyrate (BUT), as regulators of MUC proteins. For example, a cell-based study by Giromini et al. showed that treating cells with a specific concentration of BUT and SCFAs mixture for 24 h increased the mRNA levels of ZO-1, MUC2, and MUC5AC [82]. Moreover, the metabolite SCAF, which is produced by *Porphyromonas*, activates the AP1 (c-Fos/c-Jun) cis-element, increases MUC2 transcription through histone acetylation and methylation, and increases mucus production [83] and bile acids, another axis. In Apcmin/+mice, BA supplementation increased the relative abundance of *Akkermansia* and *Bacteroides*, and decreased the level of SCFAs and MUC2 expression [84]. It was mediated by STAT3 signaling to promote the progression of cancer. Additionally, tryptophan is catabolized to form indole derivatives such as indole-3-ethanol (IEt), indole-3-pyruvate (IPyA), indole-3-aldehyde (I3A), and 3-indole-propionic acid (IPA). These metabolites enhance the mucus layer by increasing MUC2 expression through activation of the AhR and PXR [85].

### 4.2. Specific Mechanisms of Key Carcinogenic Bacteria

According to the differential distribution of gut microbiota in proximal and distal CRC, as shown in Figure 1 and Table 1, we identified four important pathogenic bacteria that are closely associated with mucin expression. And they promote tumorigenesis by distinct but additive means.

#### 4.2.1. *F. nucleatum*

*F. nucleatum*, a Gram-negative anaerobic bacterium of oral origin, is known to be an opportunistic pathogen [86]. It translocates to the colorectal area by the blood or gut path and is usually discovered with a higher prevalence or abundance in the proximal than the distal colon cancers [87]. *F. nucleatum* exists in CRC, it is connected to clinical characteristics like high microsatellite instability (MSI-H), high CpG island methylator phenotype (CIMP-H), BRAF mutation, wild-type TP53, and the Consensus Molecular Subtype 1 (CMS1) [88]. These characteristics are also characteristic of proximal CRC and mucinous colorectal adenocarcinoma, but the current evidence is not sufficient to conclude that *F. nucleatum* causes these molecular profiles.

Molecularly, *F. nucleatum* uses adhesins like FadA adhesin and Fap2 lectin to adhere and invade colonic epithelial cells [89]. FadA binds to E-cadherin, a tumor suppressor that operates via β-catenin. This binding inhibits the tumor-suppressive activity of E-cadherin, causing β-catenin to be translocated into the nucleus and accumulate. This causes upregulation of oncogenes such as c-Myc, which leads to DNA damage and proliferation in many CRC cell lines [89]. At the same time, Fap2 is a lectin that recognizes and binds to D-galactose-β(1–3)-N-acetyl-D-galactosamine (Gal-GalNAc), a commonly overexpressed glycan structure in CRC [90]. The Fap2 protein also binds to the human inhibitory receptor TIGIT, which helps to protect cancer cells from being detected by natural killer (NK) cells [91]. And *F. nucleatum* LPS can activate the TLR4 signal pathway, then activate NF-kB, promoting CRC metastasis [92]. And notably, bacterial activity may result in the generation of transcription factors (TF5, ELF1), causing a decrease in the amount of miR-939-3p. This decrease subsequently upregulates FUT1, a fucosyltransferase, which leads to methylation of MUC2-related genes and silencing of MUC2 expression via this mechanism and promotes CRC [93].

#### 4.2.2. *ETBF*

*ETBF*, a subgroup of *B. fragilis* associated with distal CRC, produces the virulence factor B. fragilis toxin (BFT) [94]. BFT quickly results in a γ-secretase-driven cleavage of E-cadherin, damaging the structural and functional integrity of the colonic mucosal barrier [95]. It increases intestinal permeability and increases E-cadherin/β-catenin signaling in intestinal epithelial cells, which promotes proliferation and carcinogenesis in CRC [96]. In addition, the study also shows that the rBFT1 therapy is able to up-regulate the CCL3, CCR5, NF-kB, and TRAF-6, which can also play a role in the CCL3/CCR5, NF-kB pathway to promote the proliferation of cells and chemoresistance [97]. Additionally, the research by Jian Yang et al. found that both *ETBF* and *BFT* can activate the STAT3/ZEB2 axis, which reduces the expression of barrier-related proteins such as MUC2, occludin, and ZO-1, and promotes the expression of IL-17, leading to mucosal barrier destruction [98,99].

#### 4.2.3. *pks^+^ E. coli*

*E. coli* is a Gram-negative facultative anaerobe that is part of the healthy gut microbiota [100]. But some contain harmful elements, like *PKS^+^ E. coli* linked to CRC. The PKS island in these strains codes enzymes that produce colibactin, a genotoxic substance causing DNA damage [101,102]. Normally, intestinal epithelial cells are protected from the rest of the digestive tract by a coating of mucus, which *PKS^+^ E. coli* has a hard time penetrating. But if this mucosal barrier is breached, say by a temporary DSS treatment in mice, these bacteria can penetrate the epithelium to cause injury and chronic colitis. Additionally, *PKS^+^ E. coli* can secrete a VAT mucinase that degrades MUC2 mucin, allowing bacteria to penetrate the mucus and reach epithelial cells [79]. An APC^Min/+^ mice study showed that *pks^+^ vat^+^ E. coli* infection, but not *pks^+^ vat^-^* mutant, enhanced mucosal colonization and promoted tumorigenesis, indicating the role of VAT in promoting carcinogenesis [79]. Recent findings have added more information about these mechanisms. The bacteria directly attach to the epithelium through chaperone-usher fimbrial adhesins like FimH and FmlH; they also carry out the transfer of colibactin over long distances to other host cells via protective outer membrane vesicles (OMVs) [103,104].

#### 4.2.4. *S. gallolyticus*

*S. gallolyticus* is an opportunistic pathogen strongly associated with CRC, particularly distal CRC, and is more frequently observed in elderly and immunocompromised patients [105,106]. This bacterium has three pili loci: pil1, pil2, and pil3 [106]. Pil1 pilus promotes binding to collagen type I, and the Pil3A subunit promotes adhesion to MUC5AC mucin, which is a glycoprotein normally not found in the normal colorectum but is found in CRC [106,107]. Exploiting MUC5AC, *S. gallolyticus* accomplishes specific enrichment in colorectal tumors. Moreover, it has been shown that S. gallolyticus induces NOS2 expression, which generates nitric oxide, activates NF-κB signaling, and induces VEGF, COX-2, and proinflammatory cytokine expressions [108]. Also, the bacterium is capable of degrading gallotannins, thereby decreasing their anti-proliferative effect and thus promoting colorectal carcinogenesis [109].

## 5. Therapeutic Strategies Targeting the Gut Microbiota–Mucin Barrier Axis in CRC

**Figure 2:**
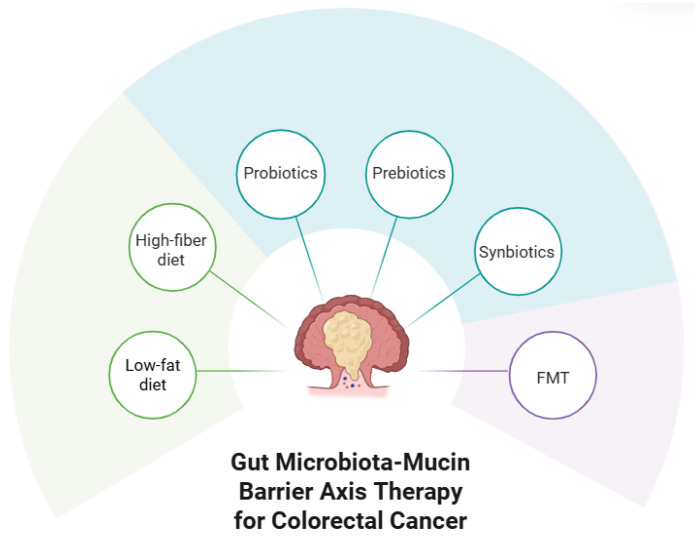
Gut microbiota–mucin barrier axis therapy for CRC.

In terms of strategies for preventing, alleviating, and maintaining the gut microbiota–mucin barrier axis, three main methods stand out: dietary intervention, microecological modulators, and fecal microbiota transplantation (FMT) (Figure 2).

### 5.1. Dietary Interventions: Foundation of Mucosal Health

CRC is considered one of the most diet-related cancers. Although genetic factors are related to only about 35% of CRC cases, environmental factors are the main cause [110]. Since gut microbiota are dependent on diet, it makes sense to alter the microbial community by favoring the beneficial bacteria over the harmful bacteria for the purpose of CRC prevention [111]. A lot of proof shows that a high-fiber diet (loaded with fruits, vegetables, and grains) is protective. It can enhance intestinal motility, improve the integrity of the intestinal barrier, and enrich beneficial gut bacteria, thus reducing the risk of CRC [112]. Dietary fiber is converted to SCFAs by fermentation in the gut, which is an essential energy source in the gut and plays a vital role in maintaining barrier function, regulating inflammation, and having anti-tumor activity [113]. Conversely, long-term high-fat diet can lead to dysbiosis of the gut microbiota [114]. Studies have shown that animal-derived monounsaturated fatty acids are positively correlated with CRC and collectively promote its development through multiple mechanisms [115]. These mechanisms include alterations in the composition of gut microbiota that reduce Paneth cell-mediated antimicrobial defenses, thereby decreasing the recruitment of dendritic cells (DCS) and the expression of MHC class II molecules in gut-associated lymphoid tissues [116]. A high-fat diet may also result in significant reductions in SCFAs in both the small intestine and feces, while promoting the proliferation of collagenase-producing microbes such as *Enterococcus faecalis*, *Proteus mirabilis*, and *Candida parapsilosis*, which are associated with tumorigenesis and recurrence [117]. Furthermore, high-fat diet-induced gut microbiota dysbiosis mediates the activation of the MCP-1/CCR2 axis, which recruits monocytes into the tumor microenvironment and promotes the polarization of tumor-associated macrophages (TAMs), thereby altering the tumor immune microenvironment [118]. Additionally, high-fat deoxygenation can accelerate increased bile secretion induced by saturated fatty acids into the intestine, where *Clostridium* converts primary bile acids into secondary bile acids; excessive exposure to bile acids can promote CRC [119,120]. Lastly, high-fat diet intake reduces the abundance of *Parabacteroides* distasonis, indirectly facilitating CRC development [121].

### 5.2. Microecological Modulators: Precision Restoration with Probiotics

Microecological modulators like probiotics, prebiotics, and synbiotics are biological agents used to regulate and improve the intestinal microecology of the host. Probiotics are live microorganisms that provide a health benefit to the host when given in sufficient quantities, improving intestinal health and increasing lifespan through the reduction in pathogenic bacteria through the lowering of the gut’s pH, competitive exclusion, and the production of antimicrobial substances [122]. Moreover, they create a variety of metabolites that improve the integrity of the intestinal barrier and lessen inflammation [123]. For example, Lactobacillus and Bifidobacterium have been clinically used and can effectively reduce chemotherapy-induced gastrointestinal complications, particularly diarrhea [124]. Next-generation probiotics (NGPs) represent a considerable turning point in the study, as they are trying to look for new probiotic strains and genetically modified microorganisms [125]. Unlike probiotics, traditionally, NGPs are more often developed for pharmaceutical purposes. For example, Liu et al. used a cell-compatible biomineralization strategy to coat BF839 with a highly resistant and detachable coating, which showed much better results in a mouse model than those without the coating, by enhancing the anti-inflammatory effect and bacterial survival greatly [126]. Another new approach was taken by Ma et al., who built a bio-mimic ‘Trojan horse’ nanoparticles Mel-SiO2 @ CCM [127]. And this nanoparticle, which was encapsulated in CRC cell membranes (CCMs) and then loaded with melittin, was able to achieve targeted drug delivery. In the mouse CRC model, this strategy significantly suppressed tumor growth by 91%, extended survival, and stimulated a tumor-specific immune response by boosting antigen presentation. Through mimicking the pathogen–host adhesion mechanism and combining natural targeting motifs to simultaneously target and kill pathogens and tumor cells, this biomimetic nanocarrier offers an innovative therapeutic strategy for *F. nucleatum*-positive CRC patients [127]. Meanwhile, prebiotics that act as “food” for probiotics are essential for maintaining gut homeostasis and exerting anti-tumor effects [128]. Substances such as fructans, inulin, resistant starch, and other oligofructoses have been proven to possess anti-cancer effects and are applied in clinical practice [129,130,131,132]. Synbiotics, which are a combination of probiotics and prebiotics, show a much more powerful synergistic effect on restoring gut microbiota balance. Ongoing studies are leading to more important roles for such microecological modulators in the prevention and treatment of cancer, offering more kinds of therapy to patients.

### 5.3. FMT: Ecosystem Resetting

FMT refers to the transfer of gut microbiota from a healthy donor to a patient’s gastrointestinal tract to correct dysbiosis and restore intestinal homeostasis. FMT has shown great success in treating recurrent *C. difficile* infection, outperforming vancomycin, and is now included in clinical guidelines [133,134]. FMT also boosts the mucosal barrier by upping the quantity of lactobacilli, augmenting butyrate metabolism, and raising the number of goblet cells and MUC2 expression [135]. In addition, in a CRC mouse model, Yu et al. also indicated that FMT promoted infiltration of anti-tumor immune cells such as CD4+ T cells and CD49b+ NK cells to the tumor microenvironment, which could increase the host anti-tumor activity through modulation of pro-inflammatory cytokine expression [136]. Currently, FMT treatment can maintain the effect of alleviating intestinal inflammation for over 1 year [137,138]. Nevertheless, it should be noted that research on FMT is still at the initial stage, and clinical trials of FMT-based therapeutics have no follow-up for the long term. Based on currently available data, we have observed transient mild adverse events following FMT treatment, including abdominal discomfort, diarrhea, constipation, and low-grade fever. Although severe side effects are rare, there appears to be some undetermined association with certain conditions, including peripheral neuropathy, idiopathic thrombocytopenic purpura, Sjögren’s syndrome, and rheumatoid arthritis [139,140,141,142]. This shows we need to know whether FMT is still safe and effective [143].

## 6. Conclusions and Future Perspectives

In summary, the gut microbiota–mucin barrier axis is a basic interface that preserves colonic homeostasis, and its dysfunction is currently recognized as an important cause of CRC pathogenesis. In this review, we discuss the complex process of mucosal barrier development and the interactions within the gut microbiota. We emphasize that pathogenic species such as *F. nucleatum*, *ETBF*, *pks+ E. coli*, and *S. gallolyticus* employ various mechanisms to disrupt mucin, thereby contributing to cancer progression. The mechanisms of action of proximal and distal microbiota are complex. Specifically, they primarily involve the direct disruption of MUC2 or MUC5AC, the induction of genotoxic damage, and the active reprogramming of host mucin gene expression. These mechanisms often overlap functionally and are currently challenging to distinguish clearly in terms of spatial localization. Looking to the future, therapeutic strategies informed by this knowledge will be propelled by integrating multi-omics datasets of both the host and microbiota, such as genomics, transcriptomics, glycomics, and proteomics. And this kind of big picture approach is going to be very important to help us get those good bacteria just right and, in the long run, to help create custom solutions and brand new insights for people who have had CRC.

## Figures and Tables

**Table 1:** Different research teams report increased or significant differences in gut microbiota between proximal and distal CRC. Consistent patterns emerge, though specific bacterial taxa vary across reports. For instance, several studies identify higher relative abundances of *Faecalibacterium*, *Blautia*, *Clostridium*, *Prevotella*, *Selenomonas*, *Lachnoclostridium*, and *Veillonella* in proximal CRC, while *Akkermansia*, *Alistipes*, *Fusobacterium*, *Escherichia/Shigella*, *Proteobacteria*, and other bacteroides species are more frequently enriched in distal CRC. These spatial differences are biologically significant, as they likely reflect distinct luminal environments, immune responses, and metabolic niches along the colon. Variations in reported taxa may arise from differences in patient cohorts, sequencing methods, and bioinformatic pipelines. Nonetheless, the consistent observation of location-specific microbial communities underscores their potential role in shaping tumor biology and clinical outcomes in CRC.

Author, Year	Proximal Bacteria	Distal Bacteria	Analytical Methods	EXP.
Flemer et al. (March 2016)	*Faecalibacterium*, *Blautia*, and *Clostridium*	*Alistipes*, *Akkermansia*, *Halomonas,* and *Shewanella*	16S rRNA sequencing	[67]
Jin et al. (September 2021)	*Acidobacteria*, *Bacteroidetes*, *Chloroflexi*, *Cyanobacteria*, *Deferribacteres*, *Elusimicrobia*, *Firmicutes*, and *Fusobacteria*	*Proteobacteria*	16S rRNA sequencing	[68]
Yang et al. (February 2018)	*Prevotella*, *Pyramido-bacterium*, *Selenomonas*, and *Peptostreptococcus*	*Fusobacterium*, *Escherichia*/*Shigella*, and *Leptotrichia*	16S rRNA sequencing	[69]
Phipps et al. (May 2021)	*Lachnoclostridium*, *Selenomonas,* and *Ruminococcus genera*	*Epsilonbacteraeota phylum*, *Campylobacteria class*, *Pasteurellales,* and *Campylobacterales orders*	16S rRNA sequencing	[70]
Liang et al. (December 2024)	*Flavonifractor plautii* (*Fp*) and *F. nucleatum*	*Bacteroides sp. A1C1 (B.A1C1) and Parvimonas micra*	16S rRNA sequencing	[71]
Lin et al. (April 2025)	*Veillonella parvula*	*Streptococcus angionosus and Peptostreptococcus anaerobius*	16S rRNA sequencing	[72]
Mouradov et al. (July 2023)	*Fusobacterium/oral pathogens*	*Firmicutes*/*Bacteroidetes, Escherichia*/*Pseudescherichia*/*Shigella*	16S rRNA sequencing	[73]
Kneis et al. (February 2023)	*Haemophilus* and *Veillonella*	*Bifidobacterium*, *Akkermansia, Roseburia*, and *Ruminococcus*	16S rRNA sequencing	[74]
Liu et al. (May 2025)	*Phocaeicola vulgatus*, *Escherichia coli*, *B.fragilis*, and *Bacteroides threoninaeae*	*Escherichia coli*, *uncultured bacterium*, and *Enterobacter kobei*	Whole-genome metagenomic sequencing	[75]

## Data Availability

No new data were created or analyzed in this study.
